# National Protocol for Model-Based Selection for Proton Therapy in Head and Neck Cancer

**DOI:** 10.14338/IJPT-20-00089.1

**Published:** 2021-06-25

**Authors:** Johannes A. Langendijk, Frank J. P. Hoebers, Martin A. de Jong, Patricia Doornaert, Chris H. J. Terhaard, Roel J. H. M. Steenbakkers, Olga Hamming-Vrieze, Jeroen B. van de Kamer, Wilko F. A. R. Verbakel, Fatma Keskin-Cambay, Johannes B. Reitsma, Arjen van der Schaaf, Liesbeth J. Boersma, Ewoud Schuit

**Affiliations:** 1 Department of Radiation Oncology, University of Groningen, University Medical Centre Groningen, Groningen, the Netherlands; 2 Department of Radiation Oncology (MAASTRO clinic), GROW School for Oncology and Developmental Biology, Maastricht University Medical Centre, the Netherlands; 3 Department of Radiation Oncology, Leiden University Medical Centre, Leiden, the Netherlands; 4 Department of Radiation Oncology, University Medical Centre Utrecht, Utrecht University, Utrecht, the Netherlands; 5 Department of Radiation Oncology, the Netherlands Cancer Institute, Amsterdam, the Netherlands; 6 Department of Radiation Oncology, Amsterdam UMC, Vrije Universiteit Amsterdam, Cancer Center Amsterdam, the Netherlands; 7 Department of Radiation Oncology, Erasmus Medical Center, Rotterdam, the Netherlands; 8 Julius Center for Health Sciences and Primary Care, University Medical Center Utrecht, Utrecht University, Utrecht, the Netherlands

**Keywords:** head and neck cancer, proton therapy, model-based selection, national indication protocol, radiation-induced side effects

## Abstract

In the Netherlands, the model-based approach is used to identify patients with head and neck cancer who may benefit most from proton therapy in terms of prevention of late radiation-induced side effects in comparison with photon therapy. To this purpose, a National Indication Protocol Proton therapy for Head and Neck Cancer patients (NIPP-HNC) was developed, which has been approved by the health care authorities. When patients qualify according to the guidelines of the NIPP-HNC, proton therapy is fully reimbursed. This article describes the procedures that were followed to develop this NIPP-HNC and provides all necessary information to introduce model-based selection for patients with head and neck cancer into routine clinical practice.

## Introduction

Radiation therapy in head and neck cancer (HNC) may result in a variety of acute and late radiation-induced toxicities significantly affecting quality of life (QoL) [1]. In the last decades, new photon-based radiation techniques, like 3-dimensional conformal radiation therapy (3DCRT) and intensity-modulated radiation therapy (IMRT), have been introduced to minimize radiation exposure to nontarget normal tissues to prevent radiation-induced toxicities. Owing to the superior beam properties of protons, radiation exposure to normal tissues can be further reduced, especially when using pencil beam scanning intensity-modulated proton therapy (IMPT) [2].

In the Netherlands, HNC patients qualify for proton therapy (PT) through the model-based approach [3, 4]. In summary, for all patients who may benefit from PT, a comparison is made between the most optimal photon and proton treatment plans. Then, normal tissue complication probability (NTCP) profiles, that is, expected toxicity rates, are estimated for both techniques. These NTCP profiles include dose distribution parameters in the organs-at-risk (OARs) and other clinical or treatment variables most relevant for the development of radiation-induced side effects. Finally, the expected clinical benefit in terms of toxicity reduction by using protons instead of photons can be calculated by subtracting the photon-based from the proton-based NTCP profile (ΔNTCP profile).

To guide model-based selection, a National Indication Protocol for PT for HNC patients (NIPP-HNC) was developed, including a detailed description of the NTCP models used for model-based selection. This article describes how the NIPP-HNC was developed and provides all information required to enable model-based selection in routine clinical practice.

## Materials and Methods

### NIPP-HNC Development Procedure

#### Working group

The NIPP-HNC was the first protocol for model-based selection for PT in the Netherlands. This section describes the procedure followed to arrive at the final version of the NIPP-HNC. A working group (WG) was formed composed of experts in the field of PT and in the field of HNC radiation therapy, with representatives from proton centers as well as centers without a formal relationship with a proton center. A clinical epidemiologist (E.S.) with special expertise in the field of clinical prediction modeling was added to the WG.

#### NTCP model selection

The first task of the WG was to select NTCP models most suitable for model-based selection. A literature search was conducted to identify papers reporting on NTCP models for radiation-induced toxicity after head and neck radiation therapy. All abstracts were reviewed by at least 2 group members and then discussed in a plenary session in which all members participated.

All papers were reviewed by using a checklist with 7 basic quality criteria as described in more detail in a previous report [3]. In addition, each model was assigned a stage of evaluation according to the TRIPOD criteria (Table 1) [5]. From these quality criteria, the WG assigned which NTCP models were considered most suitable for model-based selection. After selecting the most suitable NTCP models, an external validation and if necessary model update was performed on available original datasets.

**Table 1.** Stage of evaluation of NTCP models.

**Level**	**Description**
1a	External validation in separate dataset using *new technology in another center*
1b	External validation in separate dataset using *new technology in same center* or *same technology in another center*
2a	External validation in separate dataset using *nonrandom split* in test and validation set
2b	External validation in separate dataset using *random split* in test and validation set
3	Development and internal validation
4a	Multivariable NTCP model without internal or external validation
Level 4b	Univariable NTCP model without internal or external validation

**Abbreviation:** NTCP, Normal Tissue Complication Probability.

For this first version of the NIPP-HNC, we decided to focus only on xerostomia and dysphagia as these are the most prevalent late radiation-induced toxicities in HNC patients and as they have a significant impact on the general dimensions of QoL [1].

#### Thresholds for ΔNTCP

To arrive at a transparent and uniform system for deciding which patients will be candidates for PT, specific thresholds for ΔNTCP for the relevant toxicity outcomes needed to be agreed upon. These thresholds were established in July 2016 by the general assembly of the Netherlands Society of Radiation Oncology (NVRO) and are based on the toxicity grading according to the Common Toxicity Criteria for Adverse Events version 4.0 (CTCAEv4.0) [2, 3]. In summary, grade 1 toxicities include clinical or diagnostic observations that are asymptomatic or only lead to mild symptoms and as such were not considered relevant in model-based selection. For grade ≥ II, grade ≥ III, and grade ≥ IV toxicities, the minimal thresholds for ΔNTCP were set on ≥10%, ≥5%, and ≥2%, respectively, reflecting the increasing impact on age-appropriate instrumental activities of daily living (ADL), self-care ADL, and QoL. In case 2 or more NTCP models are used in the NIPP-HNC, patients also qualify for PT if the sum of 2 ΔNTCP values of the same toxicity grade (eg, grade ≥ II xerostomia and grade ≥ II dysphagia) are ≥15%, ≥7.5%, and ≥3%, respectively. No summation is performed across different grades. These summation thresholds were arbitrarily chosen and approved by the general assembly of the NVRO, considering the prevalence of side effects and the impact they have on patients' functioning and QoL.

The decision to use the CTCAEv4.0 as a basis for model-based selection has some drawbacks. First, as the CTCAEv4.0 is relatively new, the number of NTCP models based on this classification system will be limited. When using NTCP models for other endpoints (eg, patient-rated), the endpoint definitions should be translated to the definition of the CTCAEv4.0 as closely as possible, and a detailed description should be provided in a conversion table.

#### Procedure to approve the NIPP-HNC

Based on the literature review and additional external validation of selected models (next paragraph), a first concept NIPP-HNC was written by the NIPP-WG that was then distributed among the members of the National Platform for Proton Therapy (LPPT) and the National Platform for Head and Neck Radiotherapy (LPRHHT) for review and comments. The LPRHHT includes representatives of all radiation therapy departments in the Netherlands that treat HNC patients and is mandated to decide on site-specific guidelines for head and neck radiation therapy.

Based on the comments of the LPPT and LPRHHT, a second concept was distributed across other relevant stakeholders, such as patient advocacy groups, health insurance companies, and other relevant groups like the Dutch Society for Head and Neck Tumors and discussed during an invitational conference with these stakeholders. Based on the comments of the participants of the invitational conference, the final version was submitted to the LPRHHT for definitive approval.

The final version of the NIPP-HNC was then submitted to the National Health Care Institute (Zorginstituut Nederland, Diemen, The Netherlands). This Institute is the formal advisory organization for the Health Insurance Act. New medical interventions can only be included in the basic health care insurance package when the National Health Care Institute concludes that the scientific evidence showing a clinically relevant beneficial effect is sufficient. In October 2017, the NIPP-HNC was formally approved. Consequently, when HNC patients qualify for PT according to the guidelines of the NIPP-HNC, PT is fully reimbursed. The first patients were selected according to the guidelines of the NIPP-HNC in January 2018 and were subsequently treated with PT.

### NTCP Model Selection

#### Xerostomia

The literature search did not reveal any useful studies on models for physician-rated xerostomia according to the CTCAE. Two studies were found describing the relationship between the mean parotid dose and LENT-SOMA (Late Effects Normal Tissues – Subjective, Objective, Management, Analytic) grade ≥ 2 and RTOG (Radiation Therapy Oncology Group ) grade ≥ 3 xerostomia but the quality of these studies was regarded insufficient for either model-based selection or external validation [6, 7]. However, 7 studies were found reporting on the relationship between dose and patient-rated moderate-to-severe dry mouth as an endpoint at 3, 6, and/or 12 months after treatment (Table 2), using the EORTC QLQ-H&N35 (European Organization for Research and Treatment Quality of Life Questionnaire Head and Neck 35) [8–14]. In most studies, moderate-to-severe dry mouth was classified as a grade 3 complication, but according to the definitions of the CTCAEv4.0, this endpoint is more consistent with grade 2 (ie, moderate symptoms). From the literature review, the NIPP-WG selected the model published by Beetz et al [8] for further external validation and revision. The main reasons to select this NTCP model was as follows: (1) treatment of patients with modern radiation techniques (IMRT); (2) meticulous prospective assessment of patient-rated xerostomia with high compliance rates; (3) reasonable number of patients and events; (4) use of multivariable analysis with internal validation by means of bootstrapping; (5) good model performance; and (6) original datasets available to enable model validation and revision.

**Table 2.** Overview of studies on NTCP models for moderate-to-severe xerostomia as assessed by the EORTC QLQ-H&N35.^a^

**Reference**	**Time point, mo**	**Quality criteria for NTCP models**										**Level of evidence**
		**RT technique**	**Study design**	**Analysis**	**No. of patients**	**No. of events**	**AUC**	**Calibration tested**	**Internal validation**	**External validation**	**Individual NTCP estimation possible**	
Current study	6	Prim IMRT	Prospective	External validation	669	338	0.72	Yes	Yes	Yes	Yes	2a
Beetz 2012a [10]	6	Prim 3DCRT	Prospective	Multivariable	165	86	0.82	No	Yes	No	Yes	3
Beetz 2012b [9]	6	Prim IMRT	Prospective	External validation	161	83	0.66	Yes, poor^b^	Yes	Poor	Yes	3
Beetz 2012c [8]	6	Prim IMRT	Prospective	Multivariable	161	83	0.69	Yes	Yes	No	Yes	3
Lee 2012 [11]	12	Prim + PO IMRT	Prospective	Univariable	31	NM	0.75	Yes	No	No	Yes	4
Lee 2013 [12]	3	Prim + PO IMRT	Prospective	LKB-model	237	89	0.68	No	No	No	Yes	4
	12				146	49	0.73	No	No	No	Yes	4
Lee 2014 [13]	3	Prim + PO IMRT	Prospective	Multivariable	185	87	0.86	No	Yes	No	Yes	3
	12				117	43	0.86	No	Yes	No	Yes	3
												3
Jellema 2005 [14]	6	Prim + PO 3DCRT	Prospective	Multivariable	113	46	No	No	No	No	No	4
	12				94	35	No	No	No	No	No	4

**Abbreviations:** NTCP, Normal Tissue Complication Probability; EORTC QLQ-H&N35, European Organization for Research and Treatment Quality of Life Questionnaire Head and Neck 35; RT, radiation therapy; AUC, Area Under the Curve.; Prim, primary; IMRT, intensity-modulated radiation therapy; 3DCRT, 3-dimensional conformal radiation therapy; PO, postoperative; LKB, Lyman-Kutcher-Burman model.

a The NTCP model of Beetz 2012c [8] was ranked highest and subjected to external validation as described in this article.

b This study tried to validate the model of Beetz 2012a [10], developed in patients treated with 3DCRT, in a patient population treated with IMRT. This model performed poorly in the IMRT population, especially regarding calibration.

#### Dysphagia

Based on the qualitative criteria (Table 3), the NTCP model published by Christianen et al [15] was ranked highest for the same reasons mentioned of xerostomia. Another advantage was that the definition of the OARs involved in swallowing was described in detail in a separate article, increasing consistency of OAR delineation and reproducibility [20]. Moreover, a prospective cohort study demonstrated that swallowing-sparing IMRT using the dose parameters of this model for dose optimization actually reduced the prevalence of dysphagia grade ≥ II [16]. Another study found that this NTCP model proved to be a good predictor of the risk of dysphagia grade ≥ II after PT as well [17].

**Table 3.** Overview of studies on NTCP models for dysphagia grade II-IV according to the RTOG/EORTC Late Radiation Toxicity Scoring system.

**Reference**	**Time point, mo**	**Quality criteria for NTCP models**										**Level of evidence**
		**RT technique**	**Study design**	**Analysis**	**No. of patients**	**No. of events**	**AUC**	**Calibration tested**	**Internal validation**	**External validation**	**Individual NTCP estimation possible**	
Current study	6	Primary IMRT	Prospective	External validation	813	242	0.81	Yes	Yes	Yes	Yes	1b
Christianen 2012 [15]	6	Primary 3DCRT and IMRT	Prospective	Multivariable	354	NM	0.80	No	Yes	No	Yes	3
Christianen 2016 [16]^a^	6	SW-IMRT	Prospective	External validation	186	59	0.75	Yes	No	Yes	Yes	2a
Blanchard 2016 [17]^b^	6	Protons	Prospective	External validation	89	27	0.70	Yes	Yes	Yes	Yes	1a
Dirix 2009 [18]	Variable	Primary 3DCRT and IMRT	Cross-sectional	Multivariable	53	14	NM	No	No	No	No	4
Mazzola 2014 [19]	Acute	Primary IMRT	Retrospective	Univariable	53	46	NM	No	No	No	No	4
	3					23	NM	No	No	No	No	4
	Late					13	NM	No	No	No	No	4

**Abbreviations:** NTCP, Normal Tissue Complication Probability; RTOG/EORTC, Radiation Therapy Oncology Group / European Organization for Research and Treatment; RT, radiation therapy; AUC, Area Under the Curve; IMRT, intensity-modulated radiation therapy; 3DCRT, 3-dimensional conformal radiation therapy; NM, Not mentioned; SW-IMRT, swallowing-sparing IMRT; DVH, dose-volume histogram.

a This study used the NTCP model from Christianen 2012 [15] to guide treatment planning optimization using the DVH parameters of the NTCP model. The study showed that the observed toxicity rates were lower with SW-IMRT and that the observed toxicity rates corresponded very well with the predicted rates based on the NTCP model.

b This study externally validated the NCTP model from Christianen 2012 [15] among patients treated with protons.

#### Tube feeding dependence

In 5 studies the relationship between dose distributions in OARs and tube feeding dependence was described (Table 4) [21–25]. The endpoint in these studies was tube feeding dependence during radiation therapy, and at 6 to 12 months and beyond 12 months after completion of radiation therapy. This endpoint occurs when a patient is no longer able to maintain nutritional status without a nasal tube or percutaneous endoscopic gastrostomy tube. This endpoint corresponds to grade ≥ III (“dysphagia”) according to the CTCAEv4.0.

**Table 4.** Overview of studies on NTCP models for tube feeding dependence.

**Reference**	**Time point, mo**	**Quality criteria for NTCP models**										**Level of evidence**
		**RT technique**	**Study design**	**Analysis**	**No. of patients**	**No. of events**	**AUC**	**Calibration tested**	**Internal validation**	**External validation**	**Individual NTCP estimation possible**	
Current study	6	Primary 3DCRT and IMRT	Prospective	External validation	867	102	0.87	Yes	Yes	Yes	Yes	1b
Caudell 2010 [21]	12	IMRT	Retrospective	Univariable	83	18		No	No	No	No	4
Sanguineti 2011 [22]	During RT	IMRT	Retrospective	Multivariable	59	22		No	No	No	Yes	4
Wopken 2014 [23]	6	Primary 3DCRT and IMRT	Prospective	Multivariable	355	38	0.88	Yes	Yes	No	Yes	3
Vlacich 2014 [24]	>12	IMRT	Retrospective	Univariable	141	18		No	No	No	No	4
Li 2009 [25]	>6	IMRT	Retrospective	Univariable	39			No	No	No	No	4

**Abbreviations:** NTCP, Normal Tissue Complication Probability; RT, radiation therapy; AUC, Area Under the Curve; 3DCRT, 3-dimensional conformal radiation therapy; IMRT, intensity-modulated radiation therapy.

From the evaluation of the NIPP-WG, the NTCP model published by Wopken et al [23] was selected for further external validation for the same reasons as mentioned for xerostomia.

### External Validation and Revision of Selected Models

#### Patients

Patients were eligible for external validation in case of the following inclusion criteria: squamous cell HNC; primary tumor site in oral cavity, pharynx, and larynx; stage I-IV but M0; definitive radiation therapy with or without concomitant chemotherapy or cetuximab; treatment with IMRT or VMAT; minimal follow-up of 6 months after radiation therapy; and no previous HNC treatment. Patients had to be treated with curative intention. All NTCP models selected for external validation were previously developed at the University Medical Center Groningen. For external validation, only datasets from that center were available. Patients used for external validation were not included in previous model development studies.

All patients in the development and validation set were consecutively included in a data registration program with prospective assessment of patient, treatment, and tumor characteristics, as well as physician-rated acute and late toxicity and patient-reported outcome measures (PROMs) by means of the EORTC QLQ-C30 (European Organization for Research and Treatment Quality of Life Questionnaire C30) and EORTC QLQ-H&N35. Toxicity and PROMs were assessed at baseline, weekly during treatment, and at 6 weeks and 6 months after radiation therapy. All patients in the external validation sets were treated with photons according to the Dutch national guidelines, as described in previous reports [8, 15, 23].

#### Statistical analysis

External validation for all selected models was done retrospectively from prospectively collected data, using the close testing procedure as a basis for adjustment of the original models [26]. To account for missing values, multiple imputation was performed. For a full and more detailed description of the statistical procedure, refer to **Supplemental Data S1**.

#### Moderate-to-severe xerostomia

For a detailed description of the analysis for xerostomia, refer to **Supplemental Data S2**. This endpoint was defined as moderate-to-severe xerostomia (score 3 or 4 of question 41: “Have you had a dry mouth?” of the EORTC QLQ-H&N35) at 6 months after completion of treatment.

Patient characteristics of the development and validation cohorts are listed in **Supplemental Table S2a**. In contrast to the development population of the original model, the validation population included also patients with moderate or severe xerostomia at baseline. The original model included 2 predictors: the D_mean_ to the contralateral parotid gland and minor versus no xerostomia at baseline (XER_baseline_) (**Supplemental Table S2b**) [8].

In summary, the analysis showed that the model required model revision, meaning that the intercept and the regression coefficients of the original model had to be adjusted as shown in **Supplemental Table S2b**. Considering the relevance of xerostomia at baseline for future occurrence of xerostomia, moderate-to-severe xerostomia was added as an additional category to the XER_baseline_ predictor.

After the external validation procedure, the NTCP value for each plan and each individual patient can be calculated by the following regression formula (**RF1**):

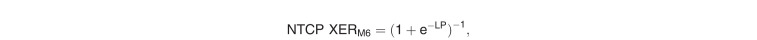
where LP = −1.507 + 0.052 × D_mean_ contralateral parotid gland

+ 0.525 × minor XER_baseline_  (1 if XER_baseline_ is minor, 0 if not)

+ 1.482 × moderate-to-severe XER_baseline  _ (1 if XER_baseline_ is moderate or severe, 0 if not).

#### Dysphagia

For a detailed description of the analysis of dysphagia, refer to **Supplemental Data S3**. Dysphagia was defined as grade II-IV dysphagia (ie, only able to eat soft or pureed food or worse) at 6 months after completion of radiation therapy (DYSPH_M6_) as assessed by health care providers.

The original model consisted of 2 prognostic factors: the D_mean_ to the superior pharyngeal constrictor muscle (PCM) and the D_mean_ to the supraglottic larynx (**Supplemental Table S3b**) [15].

The external validation set consisted of patients who had been subjected to model-based optimization, based on the original model of Christianen et al [16], and as such were different from the patients used for model development. Consequently, the D_mean_ of the superior PCM and the supraglottic area were significantly lower in the validation set than in the development set. The analysis indicated that the effect of the D_mean_ to the superior PCM increased, while the effect of D_mean_ to the supraglottic larynx decreased to almost zero, indicating that this dose parameter had no predictive value for the risk of DYSPH_M6_ in the validation cohort. Based on more recent studies showing that other predictors like the oral cavity may be an important OAR to consider, it was decided to extend the model with other predictors (**Supplemental Data S3**) [27–29]. Since the original model was developed on a small set of patients and now that more data were available to allow more reliable assessment of predictor-outcome associations, we decided to redevelop the model in a larger dataset that combined the development and validation set. This resulted in a revised model for DYSPH_M6_.

The NTCP value for each plan and each individual patient can be calculated by the following regression formula (**RF2**):

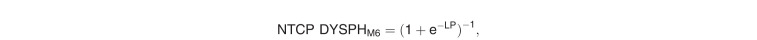
where LP = −3.303 + D_mean_ oral cavity × 0.024 + D_mean_ superior PCM × 0.024

+ baseline dysphagia × 0.967  (1 if baseline dysphagia is grade ≥ 2, 0 if not).

#### Tube feeding dependence

For a detailed description of analysis of tube feeding dependence, refer to **Supplemental Data S4**.

Tube feeding dependence was defined as not being able to maintain adequate oral intake requiring tube feeding either by nasogastric tube feeding or percutaneous endoscopic gastrostomy at 6 months after completion of treatment (TUBE_M6_).

The original model consisted of 7 prognostics factors (**Supplemental Table S4b**). The closed testing procedure revealed that model revision was required. No additional predictors were added to the model [23].

The NTCP value for each plan and each individual patient can be calculated by the following regression formula (**RF3**):

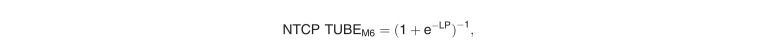
where LP = −6.849 + 0.680 × advanced T-stage  (1 if T or T4, 0 if not)

+ 0.317 × moderate weight loss  (1 if 1–10%, 0 if not)

+ 1.178 × severe weight loss  (1 if >10%, 0 if not)

+ 0.198 × accelerated radiotherapy  (1 if accelerated RT, 0 if not)

+ 1.101 × chemoradiation  (1 if chemoradiation, 0 if not)

+ 1.716 × radiotherapy plus cetuximab  (1 if RT and cetuximab, 0 if not)

+ 0.030 × D_mean_ PCM superior

+ 0.013 × D_mean_ PCM inferior

+ 0.022 × D_mean_ contralateral parotid × 0.022

+ 0.008 × D_mean_ cricopharyngeal muscle × 0.008.

### Guidelines for Model-Based Selection in the NIPP

The general eligibility criteria to qualify for PT include (1) primary tumor originating in the pharynx, larynx, or oral cavity; (2) no distant metastases; and (3) treatment with curative intent.

Based on the literature search and the subsequent external validation and model adjustment, the 3 revised NTCP models for xerostomia, dysphagia, and tube feeding dependence can be used for model-based selection. The regression formulas (RF1, RF2, and RF3) can be used to calculate the corresponding NTCP values for each individual patient.

The first step in model-based selection is to produce a photon plan with the most favorable NTCP values for the 3 toxicity endpoints (NTCP-profile-PHOTONS) while preserving conventional target coverage criteria and respecting conventional sparing of normal tissues. To this purpose, the dose-volume histogram (DVH) parameters of the 3 NTCP models can be used to guide treatment plan optimization, which is referred to as model-based plan optimization. Based on this photon plan, the NTCP-profile-PHOTONS can be calculated by using equations RF1, RF2, and RF3, respectively. From a practical point of view, it should be noted that a plan comparison is only meaningful if the minimal thresholds for ΔNTCP can potentially be reached when the dose to normal tissues is decreased to 0 Gy by using protons. So, for each toxicity endpoint, the potential maximum ΔNTCP (ΔNTCP_max_) has to be calculated first by the following equation:

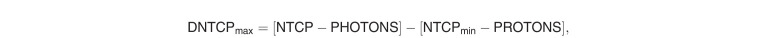
in which, NTCP_min_ − PROTONS is the NTCP value, assuming that with protons, the dose to all DVH parameters in the model can be reduced to zero.

To determine if a plan comparison is indicated and if patients eventually qualify for PT, refer to the flowchart presented in the Figure.

**Figure.**
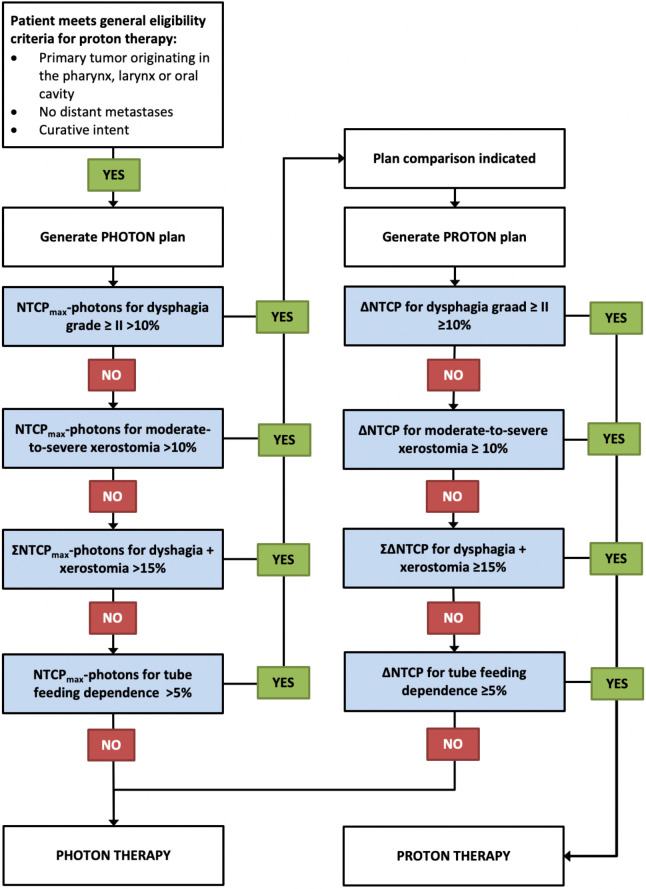
Flowchart for selecting patients for a plan comparison and PT, respectively. For each toxicity endpoint, the maximum ΔNTCP (ΔNTCP_max_) has to be calculated first by the following equation: ΔNTCP_max_ = [NTCP − PHOTONS] − [NTCP_min_ − PROTONS], in which, NTCP_min_ − PROTONS is the NTCP value, assuming that with protons, the dose to all DVH parameters in the model can be reduced to zero. Abbreviations: DVH, dose-volume histogram; NTCP, Normal Tissue Complication Probability; PT, proton therapy.

## Discussion

This article describes the procedures followed for the development of a national indication protocol for PT for HNC patients (NIPP-HNC) treated with definitive radiation therapy, with or without concurrent chemoradiation or cetuximab. The National Health Care Institute concluded that the quality of the NTCP models was sufficient for model-based selection. Consequently, when patients qualify for PT according to the guidelines of this protocol, PT is fully reimbursed.

All 3 NTCP models selected for the NIPP-HNC originated from the same center, as the literature review revealed that the models developed by this center met the quality criteria most adequately. Unfortunately, at that time no other datasets were available for external validation. Further external validation of the models in additional validation populations will help to further assess the models' external validity.

In the development and validation cohorts, patients with moderate-to-severe XER_baseline_ were excluded. Additional analysis showed that these patients did not all classify as responders at 6 months after radiation therapy and that the average D_mean_ to the contralateral parotid gland among responders was significantly higher than that in the nonresponders. Therefore, these patients were added to the combined dataset, increasing model precision and offering the possibility to extend clinical application to all patients treated with definitive radiation therapy. Only the D_mean_ of the contralateral parotid gland was identified as predictor for patient-rated xerostomia. Theoretically, it could be that these dose parameters are indeed most important for the development of patient-rated xerostomia. However, this could also be due to the problem of multicollinearity resulting from the high correlation with the D_mean_ to other salivary glands [29].

Recently, Christianen et al [16] reported on the results obtained from a subsequent cohort of 187 patients treated with swallowing-sparing IMRT, in which the D_mean_ to the superior PCM and supraglottic larynx was minimized without jeopardizing the dose to other OARs and the target volumes. That study showed good model performance in terms of discrimination and calibration, and the observed rate of DYSPH_M6_ corresponded very well with the predicted NTCP, indicating that minimizing the dose to these OARs indeed resulted in lower rates of DYSPH_M6_. Similar results were found in the current analysis with comparable discrimination and calibration. However, when the impact of the 2 dose-volume parameters were analyzed in more detail, the effect of the superior PCM was larger, but the effect of the supraglottic larynx dropped to almost zero. Consequently, the validated NTCP model would consist of only 1 dose-volume parameter of a relatively small OAR. Using such a model for dose optimization would bear the risk of pushing the dose to other anatomic sites, such as the oral cavity, while other studies indicated that the oral cavity should be considered a significant OAR, not only for dysphagia, but also for other radiation-induced side effects [18, 30–35]. It should be noted that the oral cavity was not considered when the original model was developed [2].

In the analysis of the combined cohort, the importance of the oral cavity dose as risk factor for DYSPH_M6_ was confirmed. We found a very high correlation between the superior PCM dose and oral cavity dose, which may cause multicollinearity. There are several remedies for multicollinearity, such as deleting one of the highly correlating variables. Although this would not affect the predictive power of the model, this solution was considered not optimal as the model is intended to be used for treatment optimization as well and consequently, the dose could then be pushed into the superior PCM when the oral cavity was selected and vice versa. Another remedy for multicollinearity is to combine the 2 variables into 1 merged variable, which was considered a better solution given the intended clinical utility.

In this analysis, baseline dysphagia was also identified as an independent strong predictor of DYSPH_M6_. In previous publications, patients with baseline grade ≥ 2 dysphagia were excluded. In this study, we decided not to exclude these patients, as additional analysis showed that a relatively large proportion of patients recovered at 6 months, and because recovery was highly dependent on the dose to the swallowing OARs. Moreover, the addition of baseline dysphagia to the NTCP model significantly improved model performance.

The original NTCP model, as reported by Wopken et al [23], performed reasonably in this subsequent independent validation cohort [15]. However, the closed testing procedure indicated that intercept adjustment and preferably model revision was required, which was confirmed by the fact that refitting the model in the validation cohort resulted in substantially different regression coefficients for some of the predictors in the model. This may be well explained by the fact that the number of patients with events in both cohorts was relatively low in relation to the number of predictors in the model, which may have resulted in overfitting of the original model as well as in the refitted model in the validation cohort.

This first version of the NIPP has some limitations. First, the regression coefficients of the predictors in the NTCP models used for model-based selection have some uncertainties, for instance because they are based on photon-based radiation techniques. Possibly, these models need further revision when used for patients treated with protons but also when used for photons with other optimization strategies. Consequently, NTCP models estimating the NTCP values for photon plans may differ from those for proton plans. Therefore, all patients treated with PT in the Netherlands are included in a prospective data registration program as described previously [8, 15, 23]. This so-called rapid-learning health care system enables continuous monitoring of the results obtained with PT and will serve as a screening tool to decide whether model revision is required or not. The data collected in ProTRAIT will also be used to externally validate the photon-based NTCP models that are currently used in the NIPP-HNC in patients treated with photons as well as protons and will be used to validate the clinical benefit of PT in terms of toxicity reduction according to the model-based clinical evaluation methodology [3]. A sample size calculation revealed that approximately 400 patients are required to confirm the estimated toxicity reduction with PT versus photon therapy. Given the current number of HNC patients treated with PT in the Netherlands, we expect that the first results of such a study will be available in 2022.

Second, it should be noted that all 3 endpoints concerned the assessments at 6 months after completion of treatment, as the NTCP models for these endpoints were considered most suitable for model-based selection. It could be argued that these endpoints do not reflect the toxicity severity at later time points, as they may recover at least partly over time, and thus the benefit of PT over photon therapy in the long term may be overestimated. In this regard, it should be noted that recovery of toxicity over time may occur both after proton and photon therapy and thus not necessarily affect ΔNTCP at subsequent time points. Moreover, the advantage of taking the 6-months assessment as primary endpoint is that model evaluation and revision can be performed rather quickly and results in a more dynamic rapid learning health care system.

Third, only 3 endpoints for model-based selection are used, while radiation therapy may result in a much wider range of acute and late toxicities that are not considered. Therefore, we are working on the development of comprehensive individualized toxicity risk (CITOR) profiles for HNC patients treated with radiation therapy. These CITOR profiles provide a much wider range of NTCP models for numerous acute and late toxicity endpoints during the course of radiation as well as at different time points after radiation therapy. As such, CITOR profiles may be used to produce ΔCITOR profiles, which can be regarded as biomarkers for the expected benefit of PT in terms of prevention of radiation-induced toxicity. In this regard, the current NIPP-HNC should be considered as the very first version of a dynamic document that will require further revisions in the near future.

Recently, Tambas et al [2] reported on the first experience of model-based selection in HNC. In that study, a plan comparison was made in 141 of 227 (62%) HNC patients referred for definitive radiation therapy; and using the selection procedure as described in this article, 80 patients (35%) eventually qualified for PT. Patients with advanced disease, pharyngeal tumors, and baseline complaints more frequently qualified for PT. They showed significant reductions in all DVH parameters included in the models used for model-based selection [2]. These results illustrate that model-based selection identifies patients for which the dose to relevant OARs and associated toxicity in healthy tissue can be reduced most, and as such identifies those patients who are expected to benefit most from proton compared to photon therapy.

In conclusion, this article describes the procedure followed to create the first NIPP for model-based selection for PT for HNC patients. The results presented in this article can also be used to select patients for PT in routine clinical practice.
