# Supplementary material for: National Protocol for Model-Based Selection for Proton Therapy in Head and Neck Cancer
Source: Int J Part Ther. 2021 Jun 25;8(1):354–65. doi: 10.14338/IJPT-20-00089.1 (PMC8270079; doi:10.14338/IJPT-20-00089.1)
Supplement: Supplementary file 1 [file ijpt-08-01-17_s01.docx]

# Supplementary data S1: Statistical analysis

In the development cohort, only complete cases were included, which was not the case in the validation cohort. To account for missing information regarding model predictors and endpoints in the validation set, multiple imputation was performed. Multiple imputation refers to the process of replacing missing data with substitute values and consists of three steps: 1) Fill in missing data of the incomplete data set 10 times; 2) Analysis in each of the 10 datasets, and finally: 3) Pooling of 10 analyses into one combined analysis.

The imputation model included the outcome, the predictors, and additional parameters initially considered for model development or parameters potentially related to other parameter in the imputation model, including age, gender, primary tumour site (nasopharynx vs. oropharynx vs. hypopharynx vs. larynx vs. oral cavity), target volume (local/unilateral vs. bilateral), N-stage, T-stage, baseline xerostomia, baseline dysphagia, treatment modality (conventional RT vs. accelerated RT vs. chemoradiation vs. bioradiation), D_mean_ ipsilateral and contralateral parotid glands, D_mean_ oral cavity, D_mean_ ipsilateral and contralateral submandibular gland, D_mean_ base of tongue, D_mean_ cricopharyngeal muscle, esophageal inlet muscle, superior, middle and inferior pharyngeal constrictor muscles, D_mean_ thyroid, D_mean_ glottic area, D_mean_ supraglottic area, xerostomia at 6 months, dysphagia at 6 months and tube feeding at 6 months.

The development and validation cohorts were compared for the outcome and predictor values using descriptive statistics. Then, external validation was performed by applying the original model to the individuals in the validation cohort.

Model performance was tested in terms of discrimination and calibration. Discrimination refers to the ability of the model to distinguish between patients with and without the outcome and is quantified with the c-statistic. Model calibration refers to the agreement between predicted risks and observed proportions, and was evaluated by assessing the calibration-in-the-large and calibration slope, and graphical assessment of the calibration plots. To account for multiple imputation, model performance was assessed in each imputed dataset separately. Afterwards, the c-statistic, calibration intercept and slope were pooled over imputed datasets using Rubin’s rules [**Rubin 1987**].^7^ For graphical assessment of calibration the calibration plot in the first imputed dataset was used.

A closed testing procedure was then performed to get a first indication of the need for model updating, either by recalibration-in-the-large (re-estimation of model intercept), recalibration (re-estimation of intercept and slope) or model revision (re-estimation of all coefficients) [**Vergouwe 2016**].^8^ To account for multiple imputation, the closed testing procedure was applied to each imputed dataset. To decide on the need for updating of the original model we used the majority method; i.e. the decision to update the model was based on the scenario that was most often indicated by the closed testing procedure [**Vergouwe 2010**].^9^

Depending on the results of the closed testing procedure the original model was updated or, alternatively revised in the combined cohort. Note that the latter is not part of the closed testing procedure as the closed testing procedure assumes the development data is not available to those performing the validation study.

When revising the model, the original predictors were retained but their coefficients were re-estimated depending on the results of the closed testing procedure and no other new predictors were selected. However, we allowed for the possibility of adjusting these variables, e.g. through transformation of continuous variables or changing or adding categories of categorical variables if appropriate. We assessed the possibility of a non-linear association between dose parameters and the endpoints using multifractional polynomials and restricted cubic splines plots.

To assess potential heterogeneity across the development and validation cohorts, a dummy variable for cohort was added to a no intercept model to allow for a stratified intercept per cohort (baseline risk) to assess heterogeneity in terms of baseline risk. To assess heterogeneity in predictor effects, an interaction term between the predictors and the dummy variable was added to the model. Model fit improvement was tested using ANOVA when using a stratified intercept instead of an overall intercept, or when interaction terms were added. Ideally, heterogeneity in model performance would be assessed using an internal-external cross validation.^1^ however, considering the low number of datasets available this was not considered a reliable method.

To account for potential optimism of the derived predictor effects when revising the model, a Ridge regression was applied using a penalty parameter λ derived from using a 10-fold cross-validation. Internal validity was assessed using bootstrapping techniques in which model performance was assessed in 100 bootstrap samples that were drawn with replacement. Again, all of these steps were performed within each imputed dataset separately after which decisions (e.g. non-linearity) were made based on the majority method and results were pooled using. ^2^

The statistical analysis was performed by the Julius Center in Utrecht.

1. Debray TP, Moons KG, Ahmed I, Koffijberg H, Riley RD. A framework fo developing, implementing, and evaluating clinical prediction models in an individual participant data meta-analysis. Stat Med. 2013;32(18):3158-80.
2. Rubin DB. *Multiple Imputation for Nonresponse in Surveys*. New York: J. Wiley & Sons; 1987.
