# Supplementary material for: National Protocol for Model-Based Selection for Proton Therapy in Head and Neck Cancer
Source: Int J Part Ther. 2021 Jun 25;8(1):354–65. doi: 10.14338/IJPT-20-00089.1 (PMC8270079; doi:10.14338/IJPT-20-00089.1)
Supplement: Supplementary file 2 [file ijpt-08-01-17_s02.docx]

# *Supplement S2*

# External validation xerostomia model

**Patient characteristics**

Patient characteristics are listed in **Table S2a**. The development cohort consisted of the 161 patients [**Beetz 2012c**] (**Table S2a, column A**). Information on the predictors nor the outcome was missing in the development cohort.

***Table S2a Baseline characteristics of the different patient cohorts.***

The validation cohort consisted of 508 patients. Before imputation, information on baseline xerostomia was missing in 20 patients, while information on xerostomia 6 months after treatment was missing in 120 patients because of death, lost to follow up or due to administrative failure. After imputing these missing data, seventy patients with moderate-to-severe xerostomia at baseline were excluded because the original model was not applicable to these patients. This left 438 patients for analysis in the validation cohort (**Table S2a, column B**).

*External validation*

In the imputed validation set, 200 out of 438 patients (45.7%) developed XER_m6_, which was somewhat lower than observed in the development set, in which 83 patients (51.6%) developed XER_m6_ (p=0.201).

The median D_mean_ of the contralateral parotid gland in the validation cohort was 24.2 Gy (Interquartile range (IQR) 15.3-30.7 Gy) and was somewhat lower than that in the development cohort (25.6 Gy; IQR 18.8-35.4 Gy). The distributions of D_mean_ of the contralateral parotid gland between the validation and development cohort were comparable. In the development cohort, 49 patients (30.4%) reported ‘a bit’ xerostomia at baseline compared to 131 of the evaluable patients (31.1%) in the validation cohort (p=0.874) (**Table S2a**).

The results of the external validation procedure are depicted in **Table S2b**. The calibration plots are shown in **Figure S2a**. Model performance did not improve after recalibration of the intercept only, recalibration of the slope and intercept and model revision. Because of the difference in outcome incidence between both cohort, the closed testing procedure indicated that the intercept of the original model needed updating in 8 out of 10 imputed datasets before the model could be used in the validation cohort.

The original model performed well in the validation cohort in terms of discrimination and calibration (**Table S2b**). Additional analysis using multifractional polynomials showed a non-linear relationship between D_mean-PARC_ and XER_m6_. Eventually, it was decided not to use the natural log-transformed model, based on two arguments. First, using a log-transformed dose parameter means that a small change in the low dose area has a large influence on the predicted complication risk. Consequently, the threshold for proton therapy will become substantially lower in comparison to using a regular linear transformation. Second, individuals who are planned for unilateral irradiation with the lowest risk on xerostomia will have a much higher probability to qualify for protons than those planned for bilateral irradiation with a much higher risk of xerostomia. This was considered counterintuitive and not to make sense from a clinical point of view. Therefore, the regular linear transformation was preferred and thus maintained.

***Table S2b: Model performance for the different scenarios***


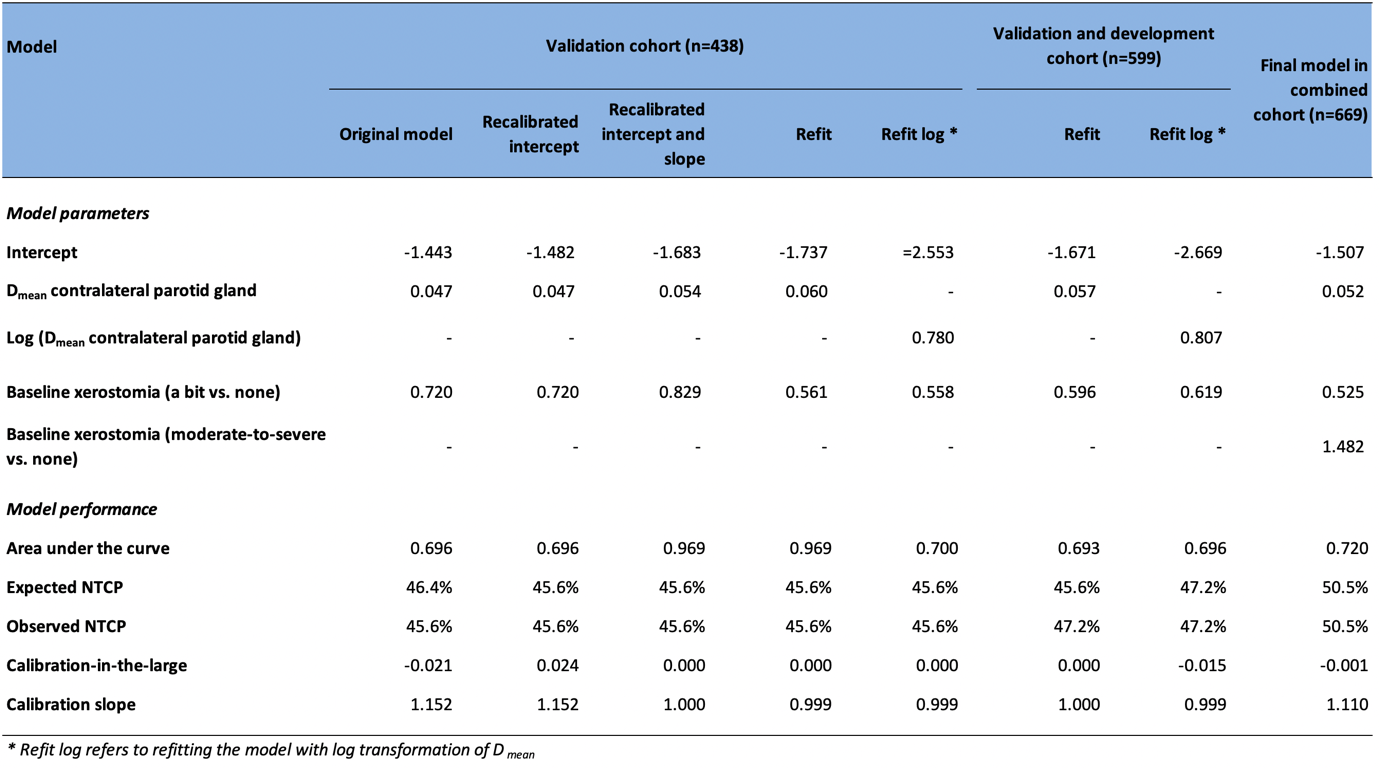


*Model revision in the combined dataset*

Given the relatively small dataset on which the original model was developed and the number of missing data in the validation set, we decided to refit the model in a combined dataset. The combined cohort (**Table S2a, column D**) was composed of the patients included in the development and validation cohorts, supplemented with patients that had moderate-to-severe xerostomia at baseline (**Table 2a, column C**). We decided to include these patients as well to increase the clinical applicability of the model and because there was a considerable number of patients that did not end up with XER_M6_ in this group. As the main objective was to externally validate the original model, only the two prognostic factors of this original model were used, and no additional candidate variables were added to nor removed from the analysis.

In this combined cohort, 338 out of 669 patients (50.5%) had XER_M6_. One hundred eighty-six (27.8%) and 70 patients (10.4%) suffered from minor xerostomia and moderate-to-severe xerostomia at baseline, respectively.

First, differences in intercept between development and validation set was assessed by adding a study indicator to the regression model. This indicated that the study indicator was not significantly associated with the outcome. An ANOVA comparing models with an overall intercept and separate intercepts per cohort also indicated a better model fit with one overall intercept (p > 0.05 in all 10 imputation sets).

When combining all variables and interaction terms in the model, no significant interactions between predictors and cohort were found. Again, using ANOVA analyses in which interaction terms were added separately indicated no significant interaction between cohort and D_mean-PARC_ and XER_baseline_ (p > 0.05 in all 10 imputed datasets). Therefore, both datasets could be pooled without accounting for any type of heterogeneity across populations.

Ridge regression was applied to each imputation set separately resulting in 10 models. In this analysis, the predictor variables were D_mean-PARC_ and baseline xerostomia (none (reference) *versus* minor *versus* moderate-to-severe). The final model was determined by pooling the estimates over the imputation sets according to Rubin’s rules. The pooled penalty parameter was 0.0186. We compared the model parameters of this final model with those of a refit made on the subset of complete cases in the validation cohort. This comparison showed that the final model was, apart from the additional XER_baseline_ category moderate-to-severe, not that different from the model that was found before using imputation.

***Figure S2 Calibration plots for the different scenarios.***

The mean predicted risk of XER_m6_ in the imputed dataset was 50.5% and corresponded well with the observed rate of 50.5%. Bootstrap validation was also performed in which the model was refitted using Ridge regression in bootstrap samples (n=100) and consecutively applied to the original combined dataset. This was done in each imputation set separately and the results were combined afterwards. This resulted in a c-statistic of 0.72 (95% CI: 0.67-0.76) with a calibration intercept of -0.001 and a calibration slope of 1.11. Based on these results, no adjustments were made to the model as the Ridge procedure already shrunk the model regression coefficients.
