# Supplementary material for: National Protocol for Model-Based Selection for Proton Therapy in Head and Neck Cancer
Source: Int J Part Ther. 2021 Jun 25;8(1):354–65. doi: 10.14338/IJPT-20-00089.1 (PMC8270079; doi:10.14338/IJPT-20-00089.1)
Supplement: Supplementary file 3 [file ijpt-08-01-17_s03.docx]

# *Supplementary data S3*

# External validation dysphagia model

**Results**

*Patient characteristics*

Patient characteristics are listed in **Table S4a**. The development cohort consisted of the 353 patients included in the original publication of Christianen, *et al* (**Table S4a, column A**).^2^ The validation cohort consisted of 354 patients, treated with SW-IMRT (**Table S4b, column B**). As information on the predictors and the outcome of the original model was available in both cohorts, there was no need for imputation of missing values.

***Table S3a: Patient characteristics in the different cohorts***

*
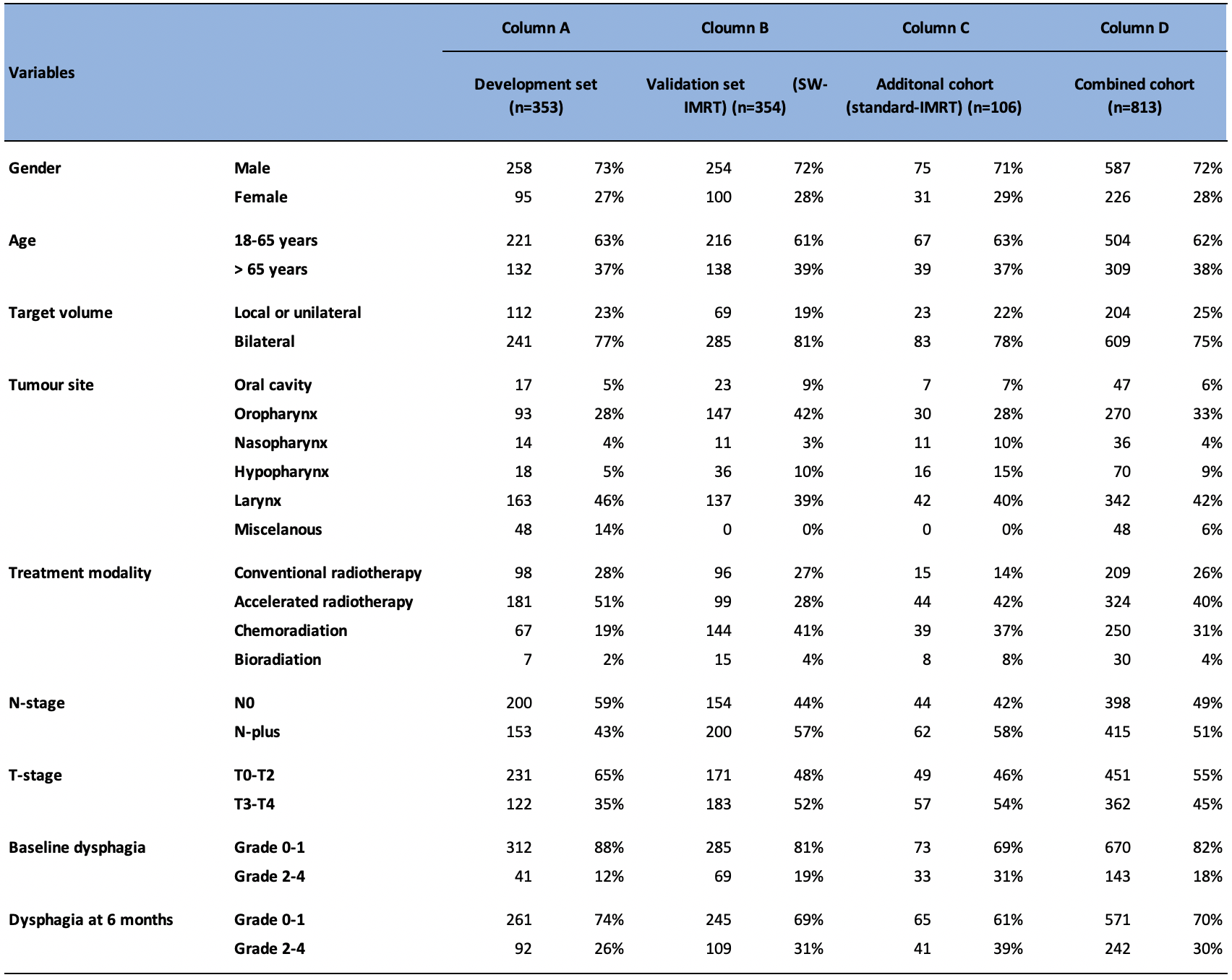
*

*External validation*

In the validation cohort, 92 out of 353 patients (26.1%) developed DYSPH_M6_, compared to 109 out of 354 patients (30.8%) in the development cohort (p=0.163).

The two cohorts differed significantly regarding the primary tumour site (P<0.001), T-stage (p<0.001), N-stage (p<0.001), target volume (p<0.001), treatment modality (p<0.001) and baseline dysphagia (p=0.003). Patients in the validation cohort had more oropharyngeal cancers, more advanced T- and N-stages and were treated more frequently with bilateral concurrent chemoradiation and less with accelerated fractionation (**Table S3a**). In addition, more patients already suffered from dysphagia at baseline in the validation cohort.

The results of the external validation procedure are depicted in **Table S3b**. The calibration plots are shown in **Figure S3**. The closed testing procedure indicated ‘model revision’, meaning that model refit in the validation population is advised. Revising the model produced regression coefficients that were substantially different from those in the original model (**Table S3b**). The revised model indicated that the effect of the mean dose to the superior PCM increased, while the effect of the supraglottic larynx decreased to almost zero, indicating that this dose parameter had no predictive value for the risk of DYSPH_M6_ in the validation cohort.

***Table S3b: Model parameters and performance for different scenarios***


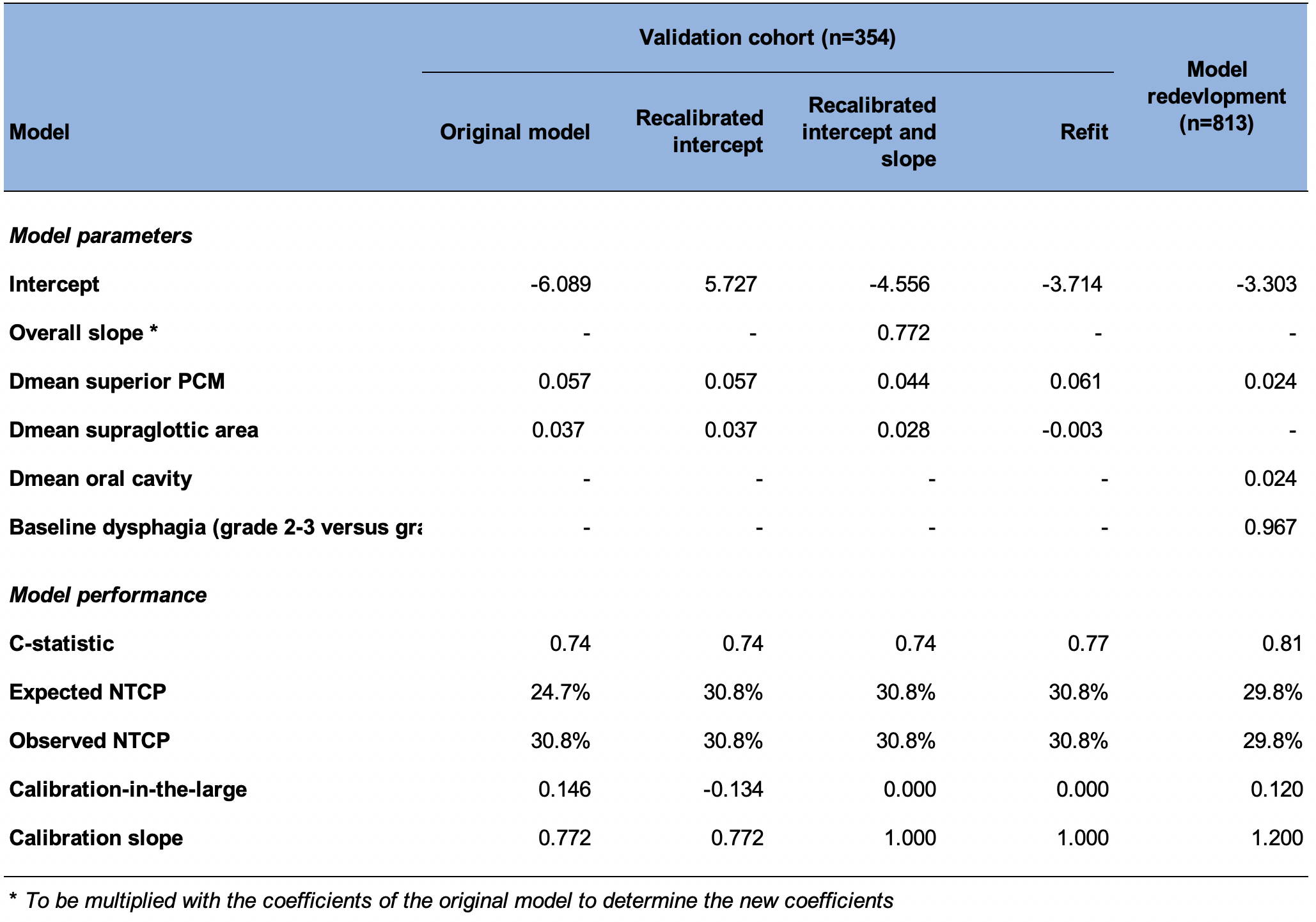


*Model extension*

The previous external validation showed that the NTCP-model needed revision. However, the revision produced a model with a very low coefficient for the mean dose to the supraglottic larynx. Consequently, only the superior PCM was left as predictor for DYSPH_M6_, which may bear the risk that when this model is used for dose optimization, the dose may be pushed away towards other yet unidentified organs-at-risk for DYSPH_M6_. This is relevant as more recent studies showed that other predictors like the oral cavity or muscles that are included in the oral cavity structure as described by Brouwer, *et al*, which was not considered in the original model as candidate variable, may be an important organ at risk as well.^1-3^ In addition, other factors like baseline dysphagia, treatment modality and tumour extension were identified as predictor for various other endpoints for swallowing dysfunction.^4-5^ As we had more data available now, in which more or better associations could be found, we decided to redevelop the model in a larger combined dataset.

***Figure S3: Calibration plots for different scenarios***

**

The combined cohort (**Table S4a, column D**) was composed of patients included in the development and validation cohort and an additional cohort of 106 patients (**Table S4a, column C**) with the same eligibility criteria but not included in either of the two other cohorts. In the development cohort, there were 227 individuals for whom the oral cavity dose was missing and could not be retrieved. This information was missing for 12 and 65 patients, respectively in both validation cohorts. Additionally, information on baseline dysphagia was missing for 6 and 2 patients, respectively. Therefore, imputation of missing data was needed. Imputation was done for all cohorts separately. After imputation, the three cohorts were combined.

To account for multiple imputation, the closed testing procedure was applied to each imputed dataset. To decide on the need for updating of the original model we used the majority method; i.e. the decision to update the model was based on the scenario that was most often indicated by the closed testing procedure.^7^

In this combined cohort, 242 out of 813 patients (29.8%) developed DYSPH_M6_. In the univariable analysis, significant associations with DYSPH_M6_ were found for gender, target volume, primary tumour site, T-stage, N-stage, baseline dysphagia and for the dose-volume the mean dose in the oral cavity, the superior and middle PCM, the supraglottic larynx, the cricopharyngeal muscle and the oesophageal inlet muscle. No significant associations were found for the salivary glands. The correlation matrix revealed a very strong correlation (r=0.92) between the mean dose to the superior PCM and the mean dose to the oral cavity. As from a physiological and functional point of view, both parameters are likely to be associated with swallowing dysfunction, we decided to combine these two dose parameters into a combined predictor which was referred to as the mean oral-cavity-superior PCM dose, which was assessed by averaging the dose to both organs.

The final multivariable analysis revealed two significant predictors for DYSPH_M6_, including the mean oral-cavity-superior-PCM dose and baseline dysphagia.

A multiple fractional polynomial analysis showed that the association between the oral-cavity-superior PCM dose and the outcome needed a non-linear transformation in the overall combined dataset (a quadratic term was indicated in 7/10 imputation sets). Further inspection revealed that this transformation was driven by the data in the development cohort. Since we judged the validation cohort to be more representative of future patients (treated with IMRT and VMAT) we decided to not transform this dose parameter and analyse its association with the outcome as a linear one. Calibration of the model was not substantially affected by this decision.

Ridge regression was applied to each imputation set separately. The final model was assessed by pooling the estimates over the imputation sets.

The mean predicted risk of DYSPH_M6_ in the imputed combined dataset was 29.8% and corresponded well with the observed rate of 29.8%. Discrimination in terms of c-statistic was 0.81 (95% CI: 0.78-0.84).

Bootstrap validation was also performed in which the model was refitted with a Ridge regression in bootstrap samples (n=100) and consecutively applied to the original combined dataset. This was done in each imputation set separately and the results were combined afterwards. The c-statistic remained similar (0.81 (95% CI: 0.78-0.84)) with a calibration intercept of 0.12 and a calibration slope of 1.20. Based on these results, no adjustments were made to the model as the Ridge procedure already shrunk the model regression coefficients.

Note that the regression coefficient for the mean dose to the oral-cavity-superior-PCM was 0.048 and that the mean dose to the oral cavity and superior PCM were split into two equally weighted separate parameters to facilitate clinical utility during treatment planning optimization.

**References**

1. Kumar R, Madanikia S, Starmer H, Yang W, Murano E, Alcorn S, McNutt T, Le Y, Quon H. Radiation dose to the floor of mouth muscles predicts swallowing complications following chemoradiation in oropharyngeal squamous cell carcinoma. Oral Oncol. 2014;50:65-70.
2. Brouwer CL, Steenbakkers RJ, Bourhis J, Budach W, Grau C, Gregoire V, van Herk M, Lee A, Maingon P, Nutting C, O'Sullivan B, Porceddu SV, Rosenthal DI, Sijtsema NM, Langendijk JA. CT-based delineation of organs at risk in the head and neck region: DAHANCA, EORTC, GORTEC, HKNPCSG, NCIC CTG, NCRI, NRG Oncology and TROG consensus guidelines. Radiother Oncol. 2015;117:83-90.
3. Schwartz DL, Hutcheson K, Barringer D, Tucker SL, Kies M, Holsinger FC, Ang KK, Morrison WH, Rosenthal DI, Garden AS, Dong L, Lewin JS. Candidate dosimetric predictors of long-term swallowing dysfunction after oropharyngeal intensity-modulated radiotherapy. Int J Radiat Oncol Biol Phys. 2010;78:1356-65.
4. Dirix P, Abbeel S, Vanstraelen B, Hermans R, Nuyts S. Dysphagia after chemoradiotherapy for head-and-neck squamous cell carcinoma: dose-effect relationships for the swallowing structures. Int J Radiat Oncol Biol Phys. 2009;75:385-92.
5. Caudell JJ, Schaner PE, Desmond RA, Meredith RF, Spencer SA, Bonner JA. Dosimetric factors associated with long-term dysphagia after definitive radiotherapy for squamous cell carcinoma of the head and neck. Int J Radiat Oncol Biol Phys. 2010;76:403-9.
