# Supplementary material for: National Protocol for Model-Based Selection for Proton Therapy in Head and Neck Cancer
Source: Int J Part Ther. 2021 Jun 25;8(1):354–65. doi: 10.14338/IJPT-20-00089.1 (PMC8270079; doi:10.14338/IJPT-20-00089.1)
Supplement: Supplementary file 4 [file ijpt-08-01-17_s04.docx]

# *Supplementary data S4*

# External validation tube feeding dependence model

*Patient characteristics*

Patient characteristics are listed in **Table S4**. The development cohort consisted of the 355 patients already included in the publication of Wopken, *et al* (**Table S4a, column A**). The initial validation cohort consisted of 534 patients. However, 22 patients were excluded because they were already tube feeding dependent at baseline. An additional 55 patients were excluded because they died before 6 months follow up, leaving 457 patients for analysis in the validation cohort (**Table S4a, column B**). After multiple imputation for other missing data, these patients were used for the external validation analysis.

*External validation*

In the validation cohort, 55 out of 457 patients (12.0%) developed TUBE_M6_, compared to 38 out of 355 patients (10.7%) in the development cohort (p=0.555).

The distributions of the D_mean-PCM-inf_ (p<0.001), D_mean-CRICO_ (p<0.001), D_mean-PARC_ (p=0.025) in the validation cohort were significantly lower in the validation cohort than in the development cohort, while the D_mean-PCM-sup_ (p=0.48) between the two cohorts was comparable. The two cohorts differed significantly regarding T-stage (p<0.001), weight loss prior to treatment (p=0.001) and treatment modality (p<0.001), Patients in the validation cohort had less advanced T-stages, had more severe weight loss and were treated more frequently with concurrent chemoradiation and conventional fractionation (**Table S4b**).

No significant heterogeneity between both cohorts was found in terms of baseline risk (intercept) and the effect of the predictors. The ANOVA comparing models with an overall intercept and separate intercepts per cohort also indicates a better model fit with one overall intercept (p > 0.05 in all 10 imputation sets).

Using ANOVA analyses in which interaction terms were added separately indicated no significant interaction between the study variable and any of the predictors (p > 0.05 in all 10 imputation sets). Therefore, both cohorts could be pooled without accounting for any type of heterogeneity across populations.

A multifractional polynomial analysis showed that the association between the dose parameters and the outcome did not necessitate a non-linear transformation in the overall combined dataset, nor in the individual datasets. This was consistent in all 10 imputed validation sets.

***Table S4a: Baseline characteristics of the validation set and development set.***

*
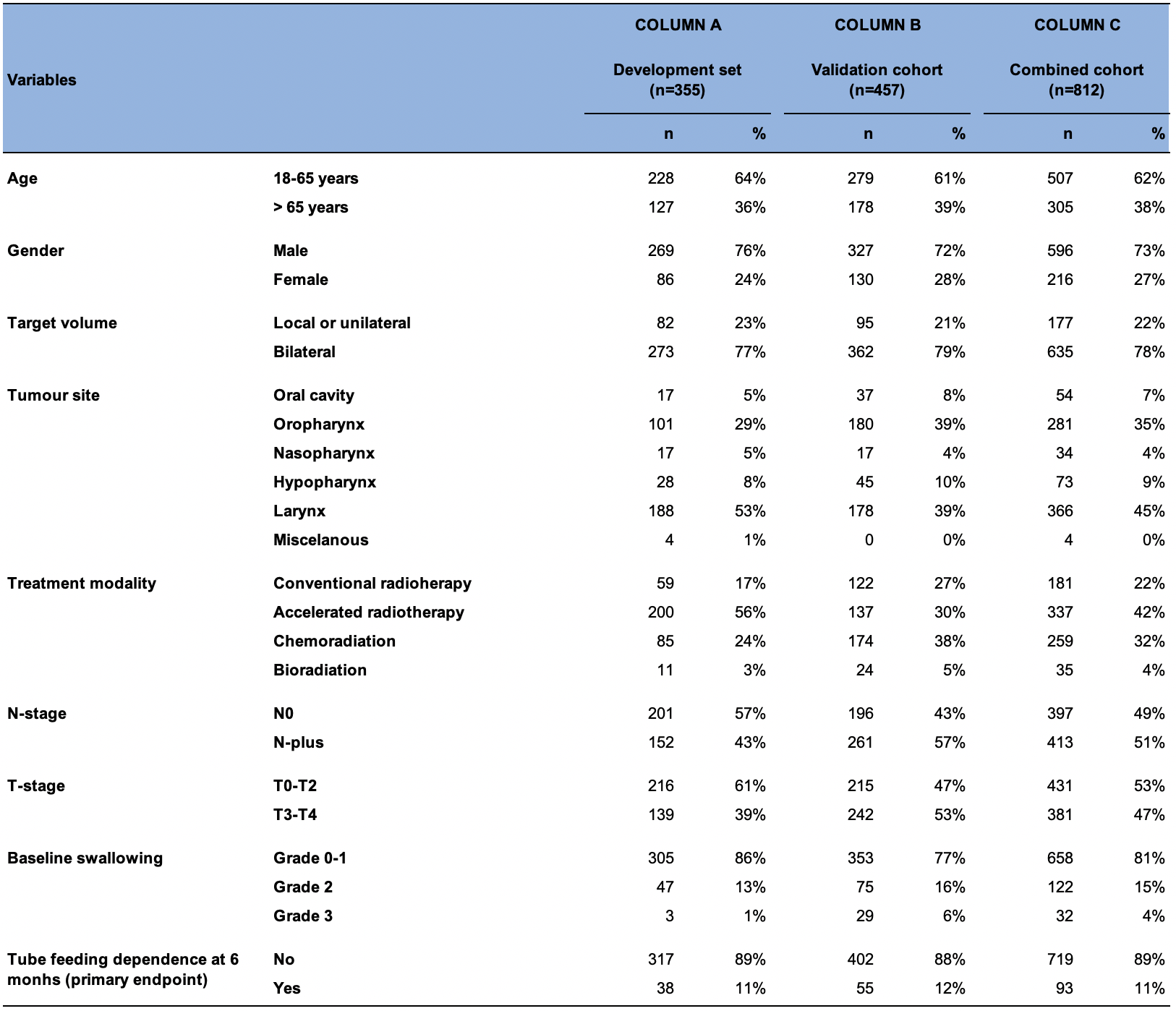
*

The results of the external validation procedure are depicted in **Table S4b**. The calibration plots are shown in **Figure S4**.

A closed testing procedure was applied to each individual imputed validation set and indicated “model with updated intercept” (5x), and “model revision” (5x), meaning that the original model should at least be adjusted in terms of the intercept and preferably refitted before its application in the validation population.

Revising the model produced regression coefficients that were substantially different from those in the original model (**Table S4b**). This may be explained by the fact that the number of patients with an event was low in relation to the number of predictors or their degrees of freedom in the model (7 variables or 10 degrees of freedom vs. 38 and 55 events in the development and validation cohort, resp.), which could potentially lead to overoptimistic models that generalize poorly to new populations.

***Table S4b: Model parameters and performance for different scenarios***

*
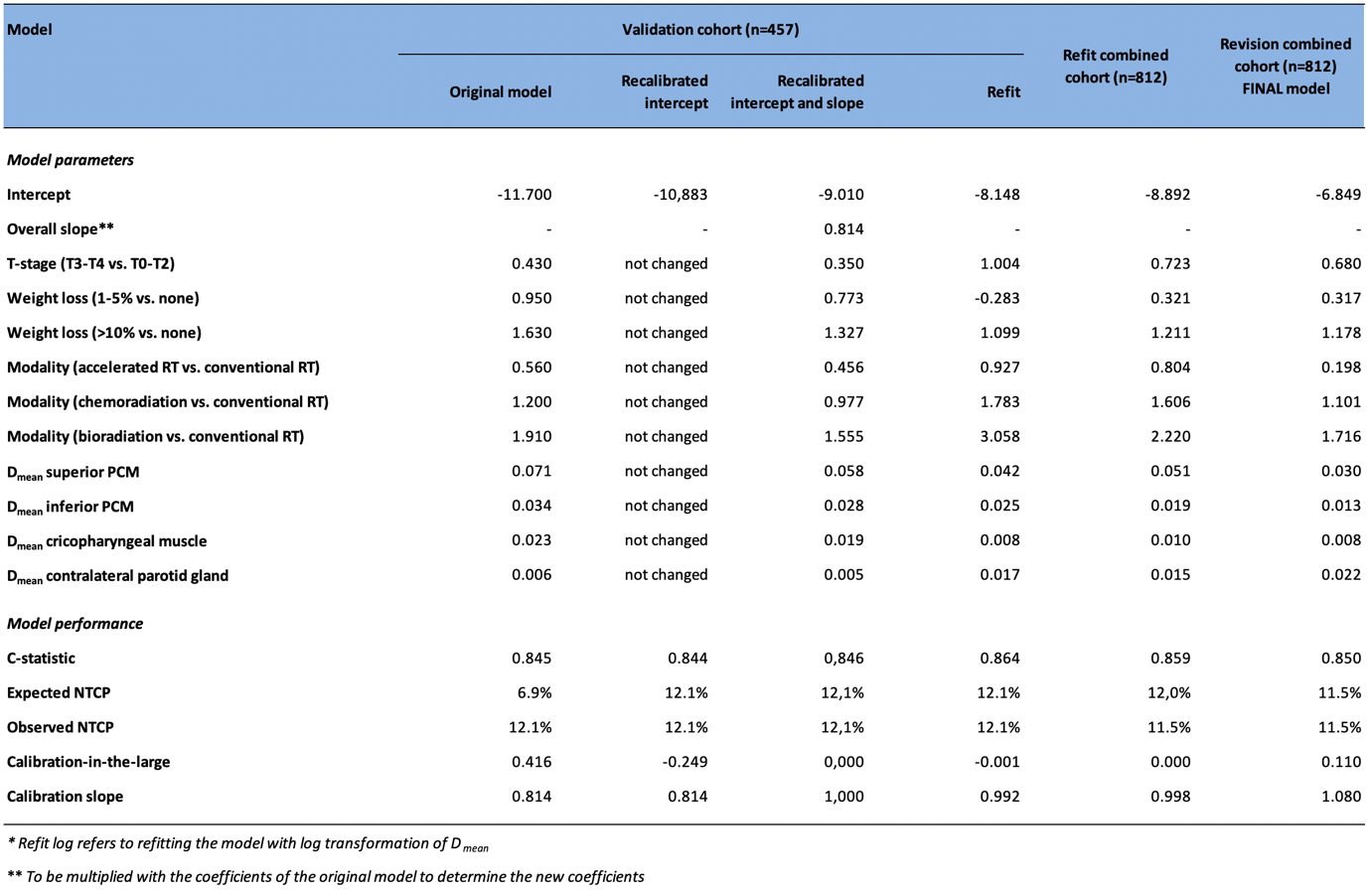
*

*Model revision in the combined dataset*

Given this relatively small number of patients with the event in both the development and validation cohorts, we decided to revise the model in the combined dataset. This revision included shrinkage, bootstrapping and imputation of missing data. As the main objective was to externally validate the original model, only the prognostic factors of this original model were used, and no additional candidate variables were added to or removed from the analysis.

The combined cohort (**Table S4a, column C**) was composed of patients included in the development and validation cohort. In this combined cohort, 93 out of 812 patients (11.5%) developed TUBE_M6_.

The mean predicted risk of TUBE_M6_ in the imputed combined dataset was 11.5% and corresponded well with the observed rate of 11.5%. Calibration plots can be found in **Figure S4**. Discrimination in terms of c-statistic was 0.86 (95% CI: 0.82-0.90). Bootstrap validation resulted in a c-statistic of 0.85 (95% CI: 0.81-0.88) with a calibration intercept of 0.11 and a calibration slope of 1.08. Based on these results, no adjustments were made to the model as the Ridge procedure already shrunk the model regression coefficients.

***Figure S4: Calibration plots for the different scenarios***
